# Genome Sequence of the Deep-Sea Denitrifier *Pseudomonas* sp. Strain MT-1, Isolated from the Mariana Trench

**DOI:** 10.1128/genomeA.01313-14

**Published:** 2014-12-18

**Authors:** Shun Fujinami, Yuji Oikawa, Takuma Araki, Yui Shinmura, Ryota Midorikawa, Hikari Ishizaka, Chiaki Kato, Koki Horikoshi, Masahiro Ito, Hideyuki Tamegai

**Affiliations:** a Bio-Nano Electronics Research Centre, Toyo University, Kawagoe, Saitama, Japan; b Department of Correlative Study in Physics and Chemistry, Graduate School of Integrated Basic Sciences, Nihon University, Setagaya-ku, Tokyo, Japan; c Department of Chemistry, College of Humanities and Sciences, Nihon University, Setagaya-ku, Tokyo, Japan; d Japan Agency for Marine-Earth Science and Technology, Yokosuka, Japan; e Faculty of Life Sciences, Toyo University, Itakura-machi, Gunma, Japan

## Abstract

*Pseudomonas* sp. strain MT-1 was the first deep-sea denitrifier isolated and characterized from mud recovered from a depth of 11,000 m in the Mariana Trench. We report here the genome sequence of this bacterium, which contributes to our understanding of denitrification and bioenergetics in the deep sea.

## GENOME ANNOUNCEMENT

*Pseudomonas* sp. strain MT-1 is a deep-sea denitrifier isolated from the Challenger Deep of the Mariana Trench (11°22.10′N 142°25.85′E, 10,898 m deep). The suitable growth conditions for MT-1 are temperatures between 4 and 45°C (optimal, 32 to 35°C), hydrostatic pressures between 0.1 and 50 MPa (optimal, 0.1 MPa), NaCl concentrations between 0 and 10% (optimal, 1 to 2%), and pH 6 to 10 (optimal, pH 7 to 8). From 16S rRNA gene analysis, MT-1 appears to be closely related to *Pseudomonas stutzeri* and *Pseudomonas aeruginosa*, but it was shown that *P. stutzeri* adapts to lower temperatures and higher hydrostatic pressures than does *P. aeruginosa* (1). MT-1 is the first denitrifying bacterium isolated from the deep sea. Therefore, by examining this bacterium, the nitrogen cycle and energy metabolism in the deep sea can be further investigated. We have identified several genes involved in denitrification and carried out additional enzymatic analyses (2–5).

The genome sequence of *Pseudomonas* sp. MT-1 contains 4,861,259 bp, with one contiguous strand. Although there are some gaps, the order of the fragments was confirmed with 8-kb paired-end sequencing. The sequence was obtained by Roche GS FLX Titanium and assembled with Celera Assembler version 5.3. The sequencing and assembly were carried out by Eurofins Genomics (Tokyo, Japan). Automatic annotation was performed using the Microbial Genome Annotation Pipeline (6), which predicted a total of 4,480 protein-coding genes. Five open reading frames (ORFs) containing a large number of gaps were removed. The names of the products for the predicted coding genes were revised manually for consistency. Fifty-seven tRNAs were predicted using the tRNAscan software (7).

As described previously, the gene sets for membrane-bound nitrate reductase (*nar*), periplasmic nitrate reductase (*nap*), and nitrous oxide reductase (*nos*) were identified in the genome of MT-1 (2–4). In the present study, all additional gene sets required for denitrification (nitrite reductase [*nir*] and nitric oxide reductase [*nor*]) were identified. The *nir* gene was found to be of the *nirS* type, consistent with previous spectroscopic analysis. These were also similar to corresponding genes of the related pseudomonads.

The annotation of the genome sequence showed that this bacterium has 22 genes encoding *c*-type cytochromes and 8 gene sets encoding aerobic respiratory terminal oxidases, suggesting that MT-1 has a highly branched aerobic respiratory chain. Further, 9 gene sets for molybdoenzymes (including Nar and Nap) were found. It is known that many anaerobic respiratory terminal enzymes, like nitrate reductase, contain molybdopterin as a cofactor. The existence of multiple molybdoenzymes suggests that *Pseudomonas* sp. strain MT-1 might utilize multiple compounds as a substrate in anaerobic respiration. These might support the growth of the bacterium in the deep-sea sediments.

### Nucleotide sequence accession numbers.

The genome sequence of *Pseudomonas* sp. strain MT-1 was deposited at DDBJ/EMBL/GenBank under the accession no. AP014655. The version described in this paper is the first version, AP014655.1.
